# The influence of constitutive law choice used to characterise atherosclerotic tissue material properties on computing stress values in human carotid plaques

**DOI:** 10.1016/j.jbiomech.2015.09.023

**Published:** 2015-11-05

**Authors:** Zhongzhao Teng, Jianmin Yuan, Jiaxuan Feng, Yongxue Zhang, Adam J. Brown, Shuo Wang, Qingsheng Lu, Jonathan H. Gillard

**Affiliations:** a Department of Radiology, University of Cambridge, UK; b Department of Engineering, University of Cambridge, UK; c Department of Vascular Surgery, Changhai Hospital, Shanghai, China; d Division of Cardiovascular Medicine, University of Cambridge, UK

**Keywords:** Strain energy density function, Atherosclerosis, Stress, Material stability, Material property

## Abstract

Calculating high stress concentration within carotid atherosclerotic plaques has been shown to be complementary to anatomical features in assessing vulnerability. Reliability of stress calculation may depend on the constitutive laws/strain energy density functions (SEDFs) used to characterize tissue material properties. Different SEDFs, including neo-Hookean, one-/two-term Ogden, Yeoh, 5-parameter Mooney–Rivlin, Demiray and modified Mooney–Rivlin, have been used to describe atherosclerotic tissue behavior. However, the capacity of SEDFs to fit experimental data and the difference in the stress calculation remains unexplored. In this study, seven SEDFs were used to fit the stress–stretch data points of media, fibrous cap, lipid and intraplaque hemorrhage/thrombus obtained from 21 human carotid plaques. Semi-analytic solution, 2D structure-only and 3D fully coupled fluid-structure interaction (FSI) analyses were used to quantify stress using different SEDFs and the related material stability examined. Results show that, except for neo-Hookean, all other six SEDFs fitted the experimental points well, with vessel stress distribution in the circumferential and radial directions being similar. 2D structural-only analysis was successful for all seven SEDFs, but 3D FSI were only possible with neo-Hookean, Demiray and modified Mooney–Rivlin models. Stresses calculated using Demiray and modified Mooney–Rivlin models were nearly identical. Further analyses indicated that the energy contours of one-/two-term Ogden and 5-parameter Mooney–Rivlin models were not strictly convex and the material stability indictors under homogeneous deformations were not always positive. In conclusion, considering the capacity in characterizing material properties and stabilities, Demiray and modified Mooney–Rivlin SEDF appear practical choices for mechanical analyses to predict the critical mechanical conditions within carotid atherosclerotic plaques.

## 1 Introduction

Carotid atherosclerotic disease is responsible for around 15–20% of all ischemic strokes (Brott et al., 2011), with the luminal stenosis being the only validated diagnostic criterion for patient risk stratification. However, this criterion becomes less reliable in patients with mild to moderate carotid stenoses (Barnett et al., 1998). Increasing evidence has suggested that both the physical characteristics of atherosclerotic plaques and the mechanical loading within the structure may allow greater potential to predict clinical progression than luminal stenosis alone. A vulnerable carotid atherosclerotic plaque is characterized by the presence of intraplaque hemorrhage (IPH) and a large lipid-rich necrotic core, with symptomatic plaques also showing evidence of fibrous cap (FC) rupture. These features have been shown to predict future events in both symptomatic (Altaf et al., 2008, Eliasziw et al., 1994) and asymptomatic (Singh et al., 2009, Takaya et al., 2006) patients. As plaques are continually subject to mechanical loading due to pulsatile blood pressure and flow, FC rupture is thought to occur when loading exceeds its material strength (Richardson et al., 1989, Tang et al., 2009a). FC stress can differentiate symptomatic from asymptomatic patients (Sadat et al., 2011, Zhu et al., 2010) and both plaque deformation and FC stress have been found to be associated with subsequent cerebrovascular ischemic events in symptomatic patients (Sadat et al., 2010, Teng et al., 2011, Teng et al., 2013).

There is therefore a need to integrate both plaque morphological and compositional features with the critical mechanical conditions for improved patient risk stratification. However, the reliability of re-predicting the critical mechanical conditions is largely dependent on the accuracy of plaque geometry and the material properties of each atherosclerotic component, including intra-plaque hemorrhage or thrombus (IPH/T), lipid and FC. Accurate reconstruction of plaque geometry is critically dependent on the imaging technique utilized, limited by resolution and tissue discrimination. The behavior of material properties is determined from experimental measurements, but also influenced by constitutive laws. Several potential strain energy density functions (SEDFs) can be used to characterize the material, such as neo-Hookean (Akyildiz et al., 2011, Caille et al., 2002, Lee et al., 1996, Ohayon and Tracqui, 2005), one-term Ogden (Barrett et al., 2009), two-term Ogden (Li et al., 2006, Li et al., 2007, Tang et al., 2008, Versluis et al., 2006), Yeoh (Cunnane et al., 2015, Lawlor et al., 2011), five-parameter Mooney–Rivlin (Gao and Long, 2008, Maher et al., 2009), Demiray (Chau et al., 2004, Delfino et al., 1997, Kaazempur-Mofrad et al., 2003a), and modified Mooney–Rivlin SEDF (Tang et al., 2009a, Tang et al., 2013, Teng et al., 2014b). These seven SEDFs have been used in numerous studies to model the mechanical behavior of carotid atherosclerotic plaques. However, the effectiveness of each SEDF at characterizing the material properties of carotid atherosclerotic tissue and the resulting variance in predicted critical mechanical conditions within the plaque structure using these different SEDFs remain unexplored.

In this study, the seven selected SEDFs are used to fit the experimental data obtained from uniaxial extension tests performed on human carotid atherosclerotic tissues. The accuracy in computing the mechanical stress within the plaque structure is assessed by using analytical solutions, idealized 2D structure-only and 3D fully coupled fluid-structure interaction (FSI) simulations and the related material stability is discussed.

## 2 Material and methods

### 2.1 Strain energy density functions

Human carotid atherosclerotic tissues exhibit non-linear stress–strain behavior at low stretch levels (Maher et al., 2009, Mulvihill et al., 2013, Teng et al., 2014c). These complexities need to be accommodated by specific SEDFs/hyperelastic material models. Several SEDFs were adopted/developed for this purpose, including neo-Hookean, one- and two-term Ogden, Yeoh, 5-parameter Mooney–Rivlin, Demiray and modified Mooney–Rivlin models, with details as follows:

***neo-Hookean model***

$$W=C_{1}\left({\bar{I}}_{1}-3\right)+\kappa \left(J-1\right)$$

***one-term Ogden model***

$$W=\frac{\mu_{1}}{\alpha_{1}}\left(\lambda_{1}^{\alpha_{1}}+\lambda_{2}^{\alpha_{1}}+\lambda_{3}^{\alpha_{1}}-3\right)+\kappa \left(J-1\right)\;$$

***two-term Ogden model***

$$W=\sum_{p=1}^{2}\frac{\mu_{p}}{\alpha_{p}}\left(\lambda_{1}^{\alpha_{p}}+\lambda_{2}^{\alpha_{p}}+\lambda_{3}^{\alpha_{p}}-3\right)+\kappa \left(J-1\right)$$

***Yeoh model***

$$W=\sum_{i=1}^{3}C_{i}{\left({\bar{I}}_{1}-3\right)}^{i}+\kappa \left(J-1\right)$$

***5-parameter Mooney–Rivlin model***

$$W=C_{10}\left({\bar{I}}_{1}-3\right)+C_{01}\left({\bar{I}}_{2}-3\right)+C_{11}\left({\bar{I}}_{1}-3\right)\left({\bar{I}}_{2}-3\right)+C_{20}{\left({\bar{I}}_{1}-3\right)}^{2}+C_{02}{\left({\bar{I}}_{2}-3\right)}^{2}+\kappa \left(J-1\right)$$

***Demiray model***

$$W=D_{1}\left[e^{D_{2}\left({\bar{I}}_{1}-3\right)}-1\right]+\kappa \left(J-1\right)$$

***modified Mooney–Rivlin model***

$$W=C_{1}\left({\bar{I}}_{1}-3\right)+D_{1}\left[e^{D_{2}\left({\bar{I}}_{1}-3\right)}-1\right]+\kappa \left(J-1\right)\;$$

Ogden material models are expressed in terms of principal stretches, $\lambda_{j}\left(j=1,2,3\right)$, while the others are expressed in terms of invariants of Cauchy–Green deformation tensor. ${\bar{I}}_{1}=J^{-2/3}I_{1}$ and ${\bar{I}}_{2}=J^{-4/3}I_{2}$ with $I_{1}$ and $I_{2}$ being the first and second invariant of the unimodular component of the left Cauchy–Green deformation tensor,

$$I_{1}=\lambda_{1}^{2}+\lambda_{2}^{2}+\lambda_{3}^{2},\;\;\;I_{2}=\lambda_{1}^{2}\lambda_{2}^{2}+\lambda_{2}^{2}\lambda_{3}^{2}+\lambda_{3}^{2}\lambda_{1}^{2}$$

in which $\lambda_{i}\;\left(i=1,2,3\right)$ is the principal stretch. J=det(F) and ***F*** is the deformation gradient. κ is the Lagrangian multiplier for the incompressibility and the remainder are material constants which will be determined by fitting experimental measurements.

### 2.2 Material testing and data fitting

**Fig. 1:**
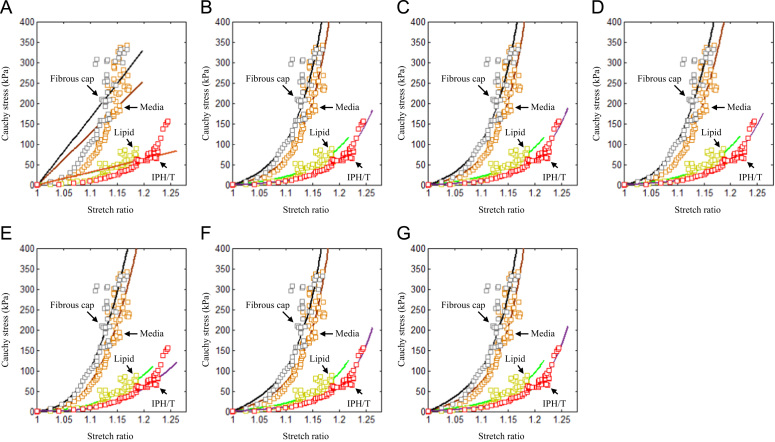
Stress–stretch data points obtained by averaging data from 21 human carotid plaque specimens, with corresponding fitted curves using different strain energy density functions. (A) neo-Hookean, (B) One-term Ogden, (C) Two-term Ogden, D) Yeoh, (E) 5-parameter Mooney-Rivlin, (F) Demiray,and (G) modified Mooney-Rivlin.

Endarterectomy carotid plaque samples from 21 symptomatic patients were collected during surgery. The local ethics committee approved the study protocol and all patients gave written informed consent. Details of tissue preparation, testing protocol and equipment used have been described previously (Teng et al., 2014c). In total, stress–stretch curves were obtained successfully from 65 media strips from 17 samples, 59 FC strips from 14 samples, 38 lipid strips from 11 samples and 21 IPH/T strips from 11 samples. An energy-based average strategy (Teng et al., 2014c) was used to obtain the representative stress–stretch curve for each atherosclerotic tissue as shown in Fig. 1 and Fig. S1 in the Supplemental material.

Cauchy stress in terms of principal stretches can be obtained from each SEDF,

(1) $$\sigma_{ii}=\lambda_{i}\frac{\partial \bar{W}}{\partial \lambda_{i}}+\kappa ,\;\left(i=1,2,3\right)$$

where $\bar{W}$ is the part in SEDFs without the incompressible term, κ(J−1). In the case of simple uniaxial extension with an incompressible tissue strip,

$$J=1,\;\lambda_{1}=\lambda ,\;\lambda_{2}=\lambda_{3}=\frac{1}{\sqrt{\lambda }}\;\mathrm{and}\;\sigma_{22}=\sigma_{33}=0$$

The stress–stretch relationship can be therefore derived,

(2) $$\sigma_{11}=\lambda_{1}\frac{\partial \bar{W}}{\partial \lambda_{1}}-\lambda_{2}\frac{\partial \bar{W}}{\partial \lambda_{2}}$$

and material constants can be obtained by minimizing the following objective function,

(3) $$S=\sum_{j=1}^{N}\left|\sigma_{11j}-\sigma_{11j}^{e}\right|$$

The relative error is used to assess the fitting quality,

$$\gamma =\frac{\sum_{j=1}^{N}\left|\sigma_{11j}-\sigma_{11j}^{e}\right|}{\sum_{j=1}^{N}\left|\sigma_{11j}^{e}\right|}\times 100\%$$

in which $\sigma_{11}$ and $\sigma_{11}^{e}$ are the predicted and measured stress, respectively; and *N* is the number of data points. In this study, all material constants were constrained to be positive as one or some negative material constants might lead to unphysical phenomena, e.g., an increased stretch leads to a decreased stress.

### 2.3 Material stability

The material stability should be taken into account when a SEDF is used to describe the material properties and to calculate mechanical conditions within the plaque. The material stability is material constant- and loading-dependent (Adina, 2013, Ogden, 2003).

Convexity is one of the criteria for assessing the material stability to some extent defined as,

(4) $$\bar{W}\prime \prime \left(\lambda_{i}\right)>0$$

implying the stress being a monotonic increasing function of the stretch ratio. If Eq. (4) holds for all $\lambda_{i}>0,\;\bar{W}$ is globally strictly convex. However, convexity depends on the measures being employed, such as stretch ratio, true strain and Green strain. Convexity in one measure does not necessarily guarantee material stability, but failure of convexity may have undesirable consequences for the development of numerical schemes (Ogden, 2003).

The material stability can also be partially characterized by using stability curves under certain loading conditions (Adina,, Adina, 2013). Considering an incompressible solid that undergoes homogeneous deformations, the equilibrium requires the equality of the external and internal virtual work as,

$$\lambda_{i}\frac{\partial \bar{W}}{\partial \lambda_{i}}+\kappa =R_{i}\lambda_{i}\;\left(\text{no}\;\text{sum}\;\text{on}\;i\right)$$

in which $R_{i}$ is the external loading. For a stable material, the work done by external loadings should always be positive, which implies the minimum eigenvalue of the matrix below must be positive,

(5) $$\left[\begin{array}{cc} K_{11}+K_{33}-2K_{13} & K_{13}+K_{33}-K_{23}-K_{13}\\ K_{21}+K_{33}-K_{23}-K_{13} & K_{22}+K_{33}-2K_{23} \end{array}\right]$$

$$K_{ij}=\lambda_{i}\lambda_{j}\frac{\partial^{2}\bar{W}}{\partial \lambda_{i}\partial \lambda_{j}}-\delta_{ij}\lambda_{i}R_{i}\;\left(\mathrm{no}\;\mathrm{sum}\;\mathrm{on}\;i\right)$$

where $\delta_{ij}$ is the Kronecker delta. The material stability can be represented by the curve of the minimum eigenvalue versus the stretch ratio or strain. Due to the involvement of external loading in the matrix, the stability curve can only be drawn under specific situations of homogeneous deformation, including uniaxial and biaxial extension and pure shear deformation.

### 2.4 Stress calculation

The difference in stress calculation using different SEDFs is quantified under the scenario of a long circular tube with a thick wall, using 2D idealized structure-only plaque models and 3D fully coupled FSI simulation with an idealized plaque geometry. The following considerations apply: there is a semi-analytic solution for calculating stress in a statically pressurized long circular tube so the result can always be obtained precisely regardless of material stability; and 2D (Kaazempur-Mofrad et al., 2003b, Li et al., 2006, Li et al., 2007, Sadat et al., 2011, Sadat et al., 2010, Zhu et al., 2010) and 3D FSI (Bluestein et al., 2008, Gao et al., 2011, Huang et al., 2014a, Leach et al., 2010, Tang et al., 2009a) analyses have been widely used in re-predicting critical mechanical conditions within carotid plaques.

#### 2.4.1 Long circular tube with a thick wall

Under static pressure, the deformation of a long tube can be solved with a plane strain assumption. The equilibrium equation and boundary conditions are,

$$\frac{d\sigma_{rr}}{dr}+\frac{\sigma_{rr}-\sigma_{\theta \theta }}{r}=0\;\mathrm{with}\;{\sigma_{rr}|}_{r=r_{o}}=0\;\mathrm{and}\;{\sigma_{rr}|}_{r=r_{i}}=-p_{i}$$

where *r*_*i*_ and *r*_*o*_ are the inner and outer radii, respectively, $\sigma_{rr}$ and $\sigma_{\theta \theta }$ are the radial and circumferential stresses, respectively and $p_{i}$ is the applied internal pressure. The pressure can be expressed in terms of stress by integrating the equilibrium equation,

(6) $$p_{i}=\int_{r_{i}}^{r_{o}}\left(\sigma_{\theta \theta }-\sigma_{rr}\right)\frac{dr}{r}$$

Therefore *r*_*i*_ can be obtained by solving the above equation numerically with the assumption of incompressibility, and stress in both circumferential and radial directions can be further computed.

#### 2.4.2 Idealized plaque models

**Fig. 2:**
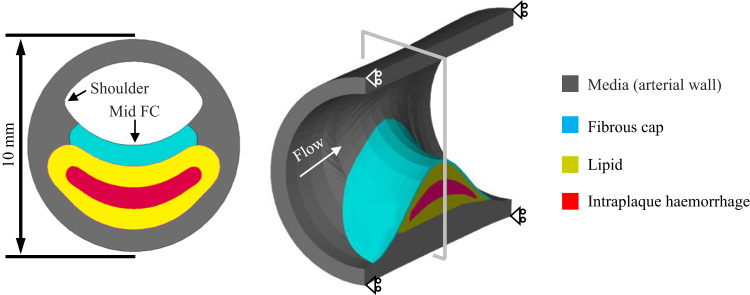
Geometry of the idealized plaque model ((A): the cross section at the most stenotic site (the 2D structure-only analysis was based on this geometry); and (B): a 3D view of the diseased section).

As shown in Fig. 2, the model represents a typical atherosclerotic plaque of 50% stenosis, composed of FC, lipid, and IPH (the rest was assumed to be media), with FC thickness being 600 µm. The stenosis of 50% was chosen to represent lesions with moderate luminal stenosis (30%–69%) for which the optimal treatment strategy remains unclear. FC thickness of 600 µm was the mean minimum value quantified by in vivo high-resolution magnetic resonance imaging in symptomatic patient groups (Sadat et al., 2009). The plaque length was set to be 20 mm and the lengths of proximal and distal section were 120 mm to avoid a potential entrance effect when FSI simulations were performed. The cross section at the most stenotic site (Fig. 2A) was used for the 2D structure-only analysis.

**Fig. 3:**
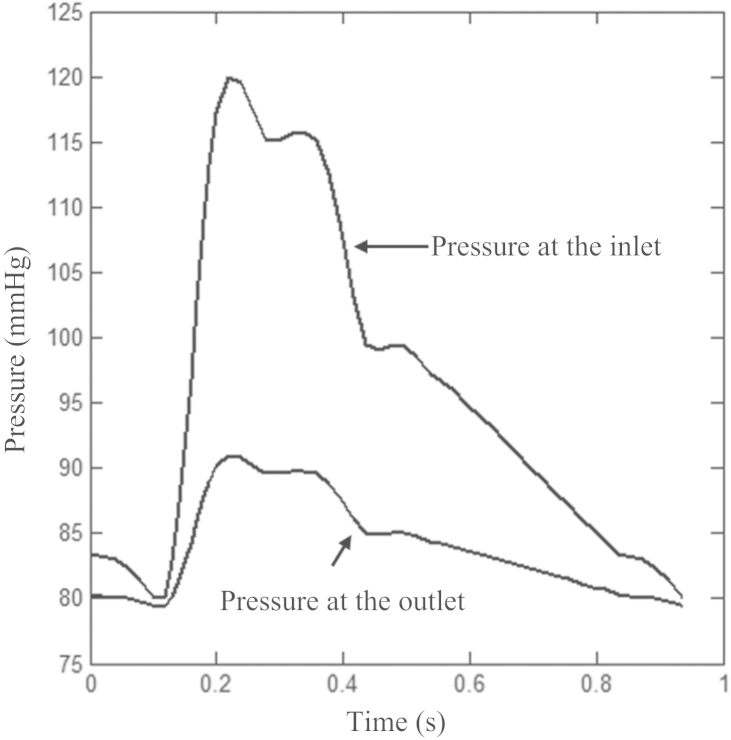
The pressure waveforms at the inlet and outlet for 3D fluid-structure interaction simulations.

For 2D models, the plaque structure was meshed using quadrilateral elements and the symmetry in geometry was considered when the fixity was applied. The pressure waveform at the inlet shown in Fig. 3 was used as the loading condition. For 3D fully coupled FSI analyses, a volume curve-fitting technique was employed, in which, the 3D plaque domain was divided into hundreds of small “volumes” to curve-fit the irregular plaque geometry with plaque component inclusions (Tang et al., 2009b). The entire plaque domain and fluid volume were meshed using hexahedral elements with 136,080 elements for the structure and 108,864 elements for the fluid, respectively. The symmetry in geometry was also considered when the fixity was applied (Fig. 2B). The pressure waveforms at both inlet and outlet are shown in Fig. 3, with systolic and diastolic pressure at the inlet being 120 and 80 mmHg, respectively. The number of time steps was set to be 200. In 3D FSI analyses, the blood flow was assumed to be Newtonian, viscous and incompressible. FSI simulations were performed using Adina 9.0.3 (Adina Inc., MA, USA). For the fluid domain, the slipping line was the central line of the fluid domain and leader–follower pairs were specified. The energy convergence criterion was used for solid domain during equilibrium iterations with the relative energy tolerance being 0.05 and relative force and moment tolerance being 0.01. For the fluid domain, the relative tolerances for velocities, pressure and displacements were set to be 0.06 to control the equilibrium. The fluid-structure coupling was solved iteratively. Both the displacement and velocities at the fluid-structure interface and the forces on the structure due to the viscous fluid were checked for convergence. Relative displacement/velocity and force tolerances were both set to be 0.06. The maximum principal stress (Stress-P_1_) over the diseased region at systole was used to characterize the critical mechanical conditions. The influence of using different SEDFs on Stress-P_1_ was subsequently analyzed.

## 3 Results

### 3.1 Stress–stretch curves fitting

As shown in Fig. 1, except for the neo-Hookean model (Fig. 1A), all SEDFs could accurately characterize the experimental data points. The detailed fitted constants for each SEDF are listed in Table 1. In addition, apart from neo-Hookean, one-term Ogden and Demiray material models, fitting results of the rest SEDFs were sensitive to initial guessed values. For example, apart from the sets of constants of the modified Mooney–Rivlin listed in Table 1, those listed in Table 2 could also well characterize the stress–stretch relationships of FC, media, Lipid and IPH/T as shown in Fig. S2 in the Supplemental material.

### 3.2 Stress distribution in the wall of a long circular tube

**Fig. 4:**
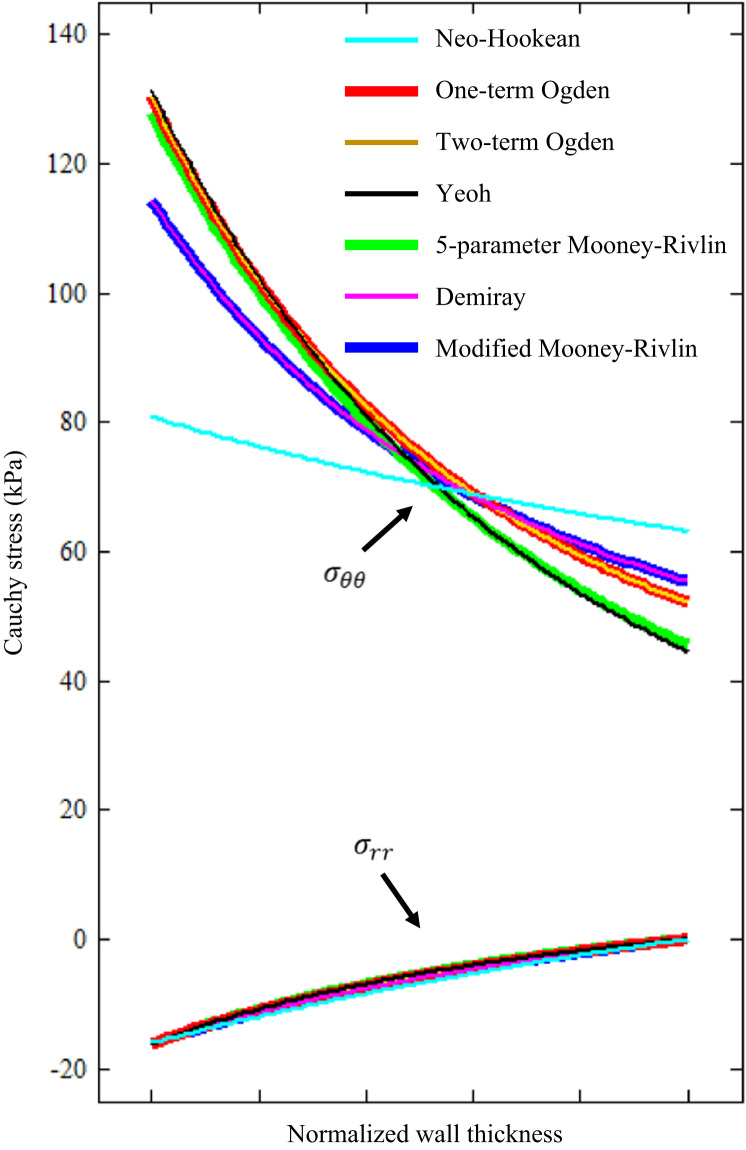
The distribution of stress in both circumferential and radial directions across the vessel wall predicted by using a semi-analytic solution with different strain energy density functions.

From a tube with the inner and outer radii of 4 and 5 mm, respectively, with internal pressure of 16 kPa, the inner and outer radii under deformed configuration can be obtained by solving Eq. (6) numerically under the assumption of plane strain. The stress distribution across the wall thickness can be computed by using Eq. (1). As shown in Fig. 4, except for the neo-Hookean model, the other six models have a similar prediction of stress in both circumferential and radial directions. The circumferential stress at the inner boundary is 80.79 for neo-Hookean, 130.36 for one-term Ogden, 130.33 for two-term-Ogden, 131.40 for Yeoh, 127.70 for 5-parameter Mooney–Rivlin, 114.33 for Demiray and 114.33 for modified Mooney–Rivlin (unit: kPa). When neo-Hookean is excluded, the variation of circumferential stress at the inner boundary was 8.3%.

### 3.3 Idealized plaque models

**Fig. 5:**
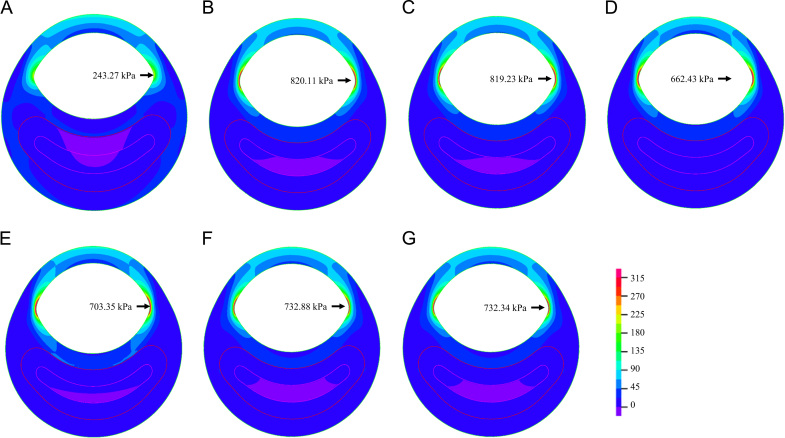
Band plots of Stress-P_1_ at systole in the 2D structure-only model using different strain energy density functions (unit: kPa). (A) neo-Hookean, (B) One-term Ogden, (C) Two-term Ogden, (D) Yeoh, (E) 5-parameter Mooney-Rivlin, (F) Demiray,and (G) Modified Mooney-Rivlin.

Differences in stress prediction became more prominent when 2D structure-only analysis with idealized plaque models were performed, as shown in Fig. 5. The stress value predicted by using neo-Hookean was the lowest (243.27 kPa), while those obtained from others were much higher (one-term Ogden: 820.11 kPa; two-term Ogden: 819.23 kPa; Yeoh: 662.43 kPa; 5-parameter Mooney–Rivlin: 703.35 kPa; Demiray: 732.88 kPa and modified Mooney–Rivlin: 732.34 kPa) with a variation of 11.1% (the value from neo-Hookean is excluded).

**Fig. 6:**
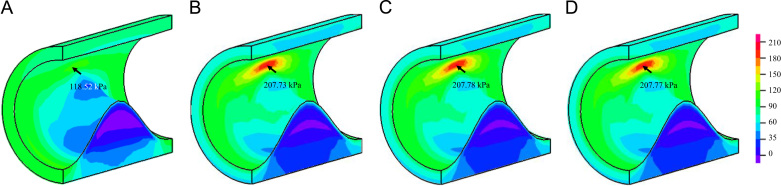
Band plots of Stress-P_1_ at systole obtained from 3D fully coupled fluid-structure interaction analyses (D: simulation results obtained using modified Mooney–Rivlin with *I*_2_ involvement; unit: kPa). (A) neo-Hookean, (B) Demiray, C) modified Mooney-Rivlin, and (D) modified Mooney-Rivlin with *I*_2_.

Consistent with previous reports, we again observed that 2D structure-only analysis overestimated the stress prediction, when compared with 3D FSI analysis (Huang et al., 2014b). As shown in Fig. 6, in 3D FSI analysis the high stress concentration in the shoulder region was much lower than that observed in 2D modeling (Fig. 5). Successful simulations were only achieved when neo-Hookean, Demiray and modified Mooney–Rivlin SEDFs were used, while the solution procedure was interrupted when a certain internal pressure loading level was reached when one-term Ogden, two-term Ogden, Yeoh and 5-parameter Mooney–Rivlin models were used.

### 3.4 Material stability

The material stability is determined by SEDF type (Adina, 2009), the relationship among material constants in each SEDF (Adina,, Ogden, 2003) and the initial-/boundary-value problems (Adina, 2013, Zheng, 2008). Considering these complexities, it is nearly impossible to validate the stability comprehensively. However, material stability can, in part, be examined by analyzing the energy contour convexity and stability curves under homogeneous deformations.

**Fig. 7:**
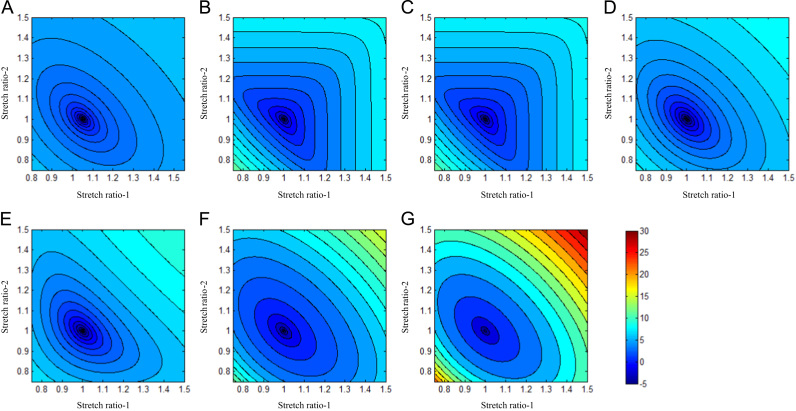
Logarithmized energy contours of fibrous cap with different strain energy density functions. (A) neo-Hookean, (B) One-term Ogden, (C) Two-term Ogden, (D) Yeoh, E) 5-parameter Mooney-Rivlin, (F) Demiray,and (G) modified Mooney-Rivlin.

**Fig. 8:**
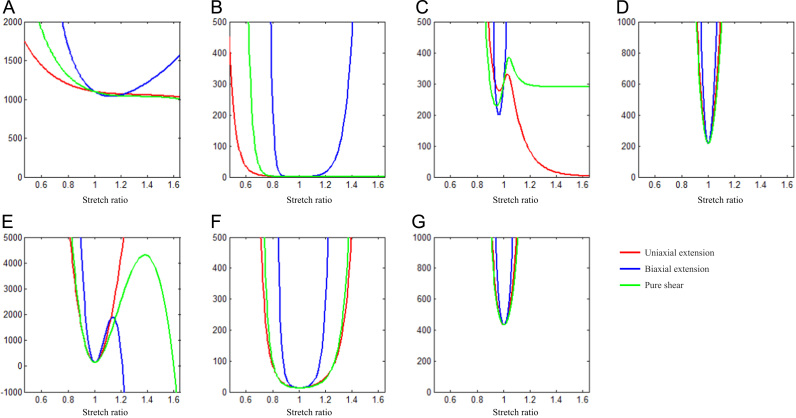
Stability curves of uniaxial extension, biaxial extension and pure shear of fibrous cap with different strain energy density functions.

As an example, the energy contour of FC is shown in Fig. 7 (those of media, lipid and IPH/T can be found in Figs. S3–S5 in the Supplemental material). The contours of neo-Hookean, Yeoh, Demiray and modified Mooney–Rivlin models are strictly convex, while those of one-term Ogden, two-term Ogden and 5-parameter Mooney–Rivlin are not. The stability curve of FC of each SEDF is shown in Fig. 8 (those of media, lipid and IPH/T are presented in Figs. S6–S8 in the Supplemental material). In certain stretch levels, the minimum eigenvalue of the matrix, shown in Eq. (5), becomes negative or approaches to zero in SEDFs of one-term Ogden, two-term Ogden and 5-parameter Mooney–Rivlin.

## 4 Discussion

Calculation of structural stress has been shown to be complementary to anatomic determinants in assessing the vulnerability of atherosclerotic (Sadat et al., 2010, Teng et al., 2014a) and aneurysmal (Khosla et al., 2014) lesions. However, the accuracy and reliability of the calculation depend on a number of factors including the resolution and tissue discrimination of the imaging modality, modeling strategy (Huang et al., 2014b), the boundary/loading conditions and the constitutive laws used to describe tissue material behavior. We believe that this is the first study to: (1) assess the difference between constitutive laws (SEDFs) in characterizing experimental data obtained from uniaxial extension tests with human carotid atherosclerotic tissue; (2) quantify the difference in stress concentrations within the plaque structure using different SEDFs; and (3) characterize the material stability of different SEDFs.

In total, seven SEDFs which had been previously used in calculating structural stress in the carotid artery were tested in this study. Results obtained indicate that, except for neo-Hooken model, all other six models could fit the experimental data appropriately (Fig. 1). However, the fitted results were sensitive to the initial guessed values for two-term Ogden, Yeoh, 5-parameter Mooney–Rivlin and modified Mooney–Rivlin models (Table 1, Table 2 and Fig. S2 in the Supplemental material). This implies that when these SEDFs are used: (1) local minimization is only reached when the objective function shown in Eq. (3) is minimized; and (2) it is difficult to interpret the physical meaning of each material constant in these SEDFs. However, such non-uniqueness appears not to have any perceptible effect on the final stress calculations. For the case of modified Mooney–Rivlin model, when the constants listed in Table 2 rather than Table 1 were used, the computed high stress concentration was 208.46 kPa, which is nearly identical as the one shown in Fig. 6C computed using constants listed in Table 1.

As listed in Table 1, except for neo-Hookean model, the relative error was similar for the same type of tissue when different SEDF was used. However, subtle differences were observed when the fitted curve and experimental data points were compared, in particular, at a low stretch range (Fig. 1). These subtle differences may result in discrepant values when predicting the stress distribution across the vessel wall when using a semi-analytic approach (Fig. 4) and the high stress concentration in the shoulder region in 2D structure-only analyses (Fig. 5). Further analyses indicated that SEDFs from the same family (Ogden: one-term and two-term Ogden models; polynomial Mooney–Rivlin: Yeoh and 5-parameter Mooney–Rivlin models; and Mooney–Rivlin with exponential term: Demiray and modified Mooney–Rivlin models) had a similar capacity in characterizing the stress–stretch behavior of each tissue type and an overall similar calculation of stress (Fig. 4, Fig. 5).

Theoretically, SEDF with any combination of stretch ratios can be developed, but not all of them can be incorporated into numerical schemes leading to a successful solution to solve initial-/boundary-value problems. In this study, successful 3D FSI simulations were obtained only with neo-Hookean, Demiray and modified Mooney–Rivlin models. The failure of one-term Ogden, two-term Ogden and 5 parameter Mooney–Rivlin models can be explained partially by the non-strictly convex energy contours (Fig. 7) and stability curves (Fig. 8). However, it is worth noting that convexity of energy contours (strain measure-dependent) and positive stability indicators (loading-dependent) do not guarantee successful 3D analyses, as exampled by Yeoh model for the idealized geometry used in this study (Fig. 2). A successful 3D fully coupled FSI analysis depends on many factors, including mesh quality, settings of time steps and interaction boundaries, etc., and it may not be reasonable to conclude that the material instability definitely accounts for the failure of the analysis with Yeoh model. However in this study, for all 3D analyses except for the difference in SEDF, all of these other factors that may influence stability were unchanged. A successful 3D structure-only analysis was achieved using Yeoh model when the pressure at the inlet (Fig. 3) was used as the loading condition applying on the entire inner surface, whilst the pressure gradient along the model was ignored. It is, therefore, possible that under a certain deformation condition, Yeoh model with material constants listed in Table 1 becomes unstable. 3D structure-only analyses with one-term Ogden, two-term Ogden and 5 parameter Mooney-Rivlin models were not successful and it was successful with neo-Hookean, Demiray and modified Mooney-Rivlin models. For those that failed in 3D analyses, the failure persisted despite efforts to adjust mesh density, time function, time steps and leader-follower settings. It should be noted that material stability under a general loading condition is complicated (Ogden, 2003) and the energy convexity shown in Fig. 7 and stability curves shown in Fig. 8 are insufficient to fully describe this characteristic.

Considering the poor capacity of neo-Hookean models to characterize the material properties of atherosclerotic tissues and the large deviations in predicting stress concentrations, this model may not be appropriate for mechanical analysis for carotid atherosclerotic plaques. Instead, both the Demiray and modified Mooney–Rivlin models may be more appropriate choices considering their capacity to characterize material properties of atherosclerotic tissues and the convergence in 2D structure-only and 3D FSI analyses. However, compared with modified Mooney–Rivlin model, Demiray model appeared to have a relatively poorer capacity in fitting experimental data from aortic tissues (Teng et al., 2015). The efficiency of modified Mooney–Rivlin models in calculating critical mechanical conditions have been validated in numerous patient-specific 2D structure-only (Sadat et al., 2010), 3D structure-only (Huang et al., 2014b, Teng et al., 2011), 3D one-way FSI (Huang et al., 2014b) and 3D full coupled FSI (Tang et al., 2009a, Teng et al., 2014b) analyses. In this study, only the first invariant of the deformation gradient, *I*_1_, was included. It is always possible to include the second invariant, *I*_2_, although the involvement of *I*_2_ did not improve the fitting quality as listed in Table 3. Moreover, the predicted stress values with *I*_2_ involvement were nearly identical in 3D FSI analyses (Fig. 6D).

**Fig. 9:**
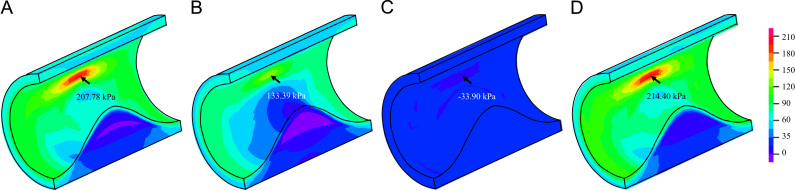
Comparisons of principal stress in different directions and von Mises stress ((A): band plot of maximum principal stress; (B): band plot of principal stress in the 2nd principal direction; (C): band plot of principal stress in the 3rd principal direction; and (D): band plot of von Mises stress).

In this study, Stress-P_1_ was used as the stress measure. The von Mises stress has also been widely used to assess the critical mechanical conditions within atherosclerotic plaques, as summarized in a recent review (Holzapfel et al., 2014). The von Mises stress, $\sigma_{v}$, is an important measure charactering material yielding due to excessive shear stresses,

$$\sigma_{v}=\sqrt{\frac{1}{2}\left[{\left(\sigma_{1}-\sigma_{2}\right)}^{2}+{\left(\sigma_{2}-\sigma_{3}\right)}^{2}+{\left(\sigma_{3}-\sigma_{1}\right)}^{2}\right]}$$

in which $\sigma_{i}$ (*i*=1, 2, 3) stands for the principal stress in the *i*th direction and Stress-P_1_=max [$\sigma_{i}$ (*i*=1, 2, 3)]. In the idealized plaque model used in this study, the distribution of Stress-P_1_ and von Mises stress was similar with less than 5% difference in calculating the high stress concentration in the shoulder region (Fig. 9). In general, Stress-P_1_ is often used to assess the failure of brittle materials and may better govern plaque structure failure, as tensile and compressive stresses frequently coexist due to geometrical complexity (Fig. 9A and C). However, debate continues regarding which stress measure is optimal to characterize the critical mechanical conditions within an atherosclerotic plaque (Holzapfel et al., 2014).

There are some limitations of the current study, (1) atherosclerotic tissues are fiber-oriented and their anisotropic material properties were not considered. This may result in the stress levels reported in this study being underestimated; (2) no attempt was made to model residual stresses and this may lead to an overestimation of stress levels (Delfino et al., 1997); (3) calcium was not included in idealized plaque models; (4) the convergence of FSI depends on geometry, mesh, settings of loading steps and interaction boundaries, amongst other parameters. Thus, it is virtually impossible to ensure that failure was due to material instability alone, excluding all other factors; and (5) different commercial numerical packages may use different strategies to solve linear equations and handle the iteration and convergence. The conclusions obtained in this study are based on Adina 9.0.3 (Adina Inc., MA, USA) and may not be valid when other numerical packages are used.

## Disclosure

The authors do not have any conflict of interest to be declared.

## Figures and Tables

**Table 1:** Fitted constants of atherosclerotic tissues using different material models.

Material model	Tissue type	Constants					*γ* (%)	Initial guessed value sensitive?
neo-Hookean		$C_{1}$ (kPa)						No
$W=C_{1}\left(I_{1}-3\right)+\kappa \left(J-1\right)$	FC	274.432					25.6	
Media	210.986					28.1	
Lipid	50.537					25.6	
IPH/T	51.675					29.2	
one-term Ogden		$\mu_{1}$ (kPa)	$\alpha_{1}$					No
$W=\frac{\mu_{1}}{\alpha_{1}}\left(\lambda_{1}^{\alpha_{1}}+\lambda_{2}^{\alpha_{1}}+\lambda_{3}^{\alpha_{1}}-3\right)+\kappa \left(J-1\right)$	FC	13.922	21.817				17.3	
Media	6.826	24.496				18.3	
Lipid	4.748	16.335				19.7	
IPH/T	1.427	20.851				10.4	
two-term Ogden		$\mu_{1}$ (kPa)	$\alpha_{1}$	$\mu_{2}$	$\alpha_{2}$			Yes
$W=\sum_{p=1}^{2}\frac{\mu_{p}}{\alpha_{p}}\left(\lambda_{1}^{\alpha_{p}}+\lambda_{2}^{\alpha_{p}}+\lambda_{3}^{\alpha_{p}}-3\right)+\kappa \left(J-1\right)$	FC	0.150	22.874	13.773	21.804		17.3	
Media	1.272	25.794	5.583	24.127		14.4	
Lipid	2.030	1.843	4.514	16.548		19.7	
IPH/T	0.549	23.971	1.324	14.877		10.3	
Yeoh		$C_{1}$ (kPa)	$C_{2}$ (kPa)	$C_{3}$ (kPa)				Yes
$W=\sum_{i=1}^{3}C_{i}{\left(I_{1}-3\right)}^{i}+\kappa \left(J-1\right)$	FC	53.724	2201.011	42.551			16.3	
Media	5.636	1816.773	162.037			17.1	
Lipid	18.548	207.371	422.652			19.6	
IPH/T	11.225	69.214	781.546			10.7	
5-parameter Mooney–Rivlin		$C_{10}$ (kPa)	$C_{01}$ (kPa)	$C_{11}$ (kPa)	$C_{20}$ (kPa)	$C_{02}$ (kPa)		Yes
$W=C_{10}\left({\bar{I}}_{1}-3\right)+C_{01}\left({\bar{I}}_{2}-3\right)+C_{11}\left({\bar{I}}_{1}-3\right)\left({\bar{I}}_{2}-3\right)+C_{20}{\left({\bar{I}}_{1}-3\right)}^{2}+C_{02}{\left({\bar{I}}_{2}-3\right)}^{2}+\kappa \left(J-1\right)$	FC	28.499	8.634	56.755	150.483	2721.008	16.1	
Media	9.267	3.508	1183.007	305.463	504.507	17.2	
Lipid	5.162	4.317	5.886	6.390	402.562	18.9	
IPH/T	0.662	0.091	21.177	228.249	2.512	11.9	
Demiray		$D_{1}$ (kPa)	$D_{2}$					No
$W=D_{1}\left[e^{D_{2}\left(I_{1}-3\right)}-1\right]+\kappa \left(J-1\right)$	FC	6.217	17.444				18.1	
Media	3.841	18.789				19.1	
Lipid	2.466	10.466				20.2	
IPH/T	0.976	13.007				10.0	
modified Mooney–Rivlin		$C_{1}$ (kPa)	$D_{1}$ (kPa)	$D_{2}$				Yes
$W=C_{1}\left(I_{1}-3\right)+D_{1}\left[e^{D_{2}\left(I_{1}-3\right)}-1\right]+\kappa \left(J-1\right)$	FC	0.130	6.208	17.452			18.1	
Media	0.138	3.832	18.803			19.1	
Lipid	0.049	2.460	10.477			20.2	
IPH/T	2.779	0.787	13.861			10.0	

**Table 2:** An alternative set of constants obtained based on different guessed values also could fit the experimental data points well when the Modified Mooney–Rivlin model was used.

Tissue type	$C_{1}$ (kPa)	$D_{1}$ (kPa)	$D_{2}$	γ (%)
FC	7.316	5.737	17.923	18.2
Media	0.527	3.808	18.843	19.1
Lipid	5.246	1.971	11.244	20.3
IPH/T	12.896	0.305	17.918	9.8

**Table 3:** The fitted material parameters when *I*_2_ was included in the modified Mooney–Rivlin model.

Tissue type	$C_{1}$ (kPa)	$C_{2}$ (kPa)	$D_{1}$ (kPa)	$D_{2}$	*γ* (%)
FC	0.242	3.522	5.986	17.673	18.2
Media	1.208	0.064	3.763	18.920	19.1
Lipid	0.008	0.417	2.415	10.551	20.2
IPH/T	4.846	0.243	0.623	14.890	9.9

## Footnotes

Appendix A Supplementary data associated with this article can be found in the online version at 10.1016/j.jbiomech.2015.09.023.

## References

[bib1] Adina. Instability of two-term Mooney–Rivlin model. 〈http://www.adina.com/newsgh48.shtml〉, 2009.

[bib2] Adina, 2013. Adina theory and modeling guide. Adina Solids & Structures, vol. I. Adina R&D, Inc Watertown, MA, USA 579 584

[bib3] Akyildiz A.C., Speelman L., van Brummelen H., Gutiérrez M.A., Virmani R., van der Lugt A., Van Der Steen A., Wentzel J.J., Gijsen F. (2011). Effects of intima stiffness and plaque morphology on peak cap stress. Biomed. Eng. Online.

[bib4] Altaf N., Daniels L., Morgan P.S., Auer D., MacSweeney S.T., Moody A.R., Gladman J.R. (2008). Detection of intraplaque hemorrhage by magnetic resonance imaging in symptomatic patients with mild to moderate carotid stenosis predicts recurrent neurological events. J. Vasc. Surg..

[bib5] Barnett H.J., Taylor D.W., Eliasziw M., Fox A.J., Ferguson G.G., Haynes R.B., Rankin R.N., Clagett G.P., Hachinski V.C., Sackett D.L., Thorpe K.E., Meldrum H.E., Spence J.D. (1998). Benefit of carotid endarterectomy in patients with symptomatic moderate or severe stenosis. North American symptomatic carotid endarterectomy trial collaborators. New Engl. J. Med..

[bib6] Barrett S., Sutcliffe M., Howarth S., Li Z.Y., Gillard J. (2009). Experimental measurement of the mechanical properties of carotid atherothrombotic plaque fibrous cap. J. Biomech..

[bib7] Bluestein D., Alemu Y., Avrahami I., Gharib M., Dumont K., Ricotta J.J., Einav S. (2008). Influence of microcalcifications on vulnerable plaque mechanics using fsi modeling. J. Biomech..

[bib8] Brott T.G., Halperin J.L., Abbara S., Bacharach J.M., Barr J.D., Bush R.L., Cates C.U., Creager M.A., Fowler S.B., Friday G., Hertzberg V.S., McIff E.B., Moore W.S., Panagos P.D., Riles T.S., Rosenwasser R.H., Taylor A.J. (2011). 2011 asa/accf/aha/aann/aans/acr/asnr/cns/saip/scai/sir/snis/svm/svs guideline on the management of patients with extracranial carotid and vertebral artery disease. J. Am. Coll. Cardiol..

[bib9] Caille N., Thoumine O., Tardy Y., Meister J.J. (2002). Contribution of the nucleus to the mechanical properties of endothelial cells. J. Biomech..

[bib10] Chau A.H., Chan R.C., Shishkov M., MacNeill B., Iftimia N., Tearney G.J., Kamm R.D., Bouma B.E., Kaazempur-Mofrad M.R. (2004). Mechanical analysis of atherosclerotic plaques based on optical coherence tomography. Ann. Biomed. Eng..

[bib11] Cunnane E.M., Mulvihill J.J., Barrett H.E., Walsh M.T. (2015). Simulation of human atherosclerotic femoral plaque tissue: the influence of plaque material model on numerical results. Biomed. Eng. Online.

[bib12] Delfino A., Stergiopulos N., Moore J.E., Meister J.J. (1997). Residual strain effects on the stress field in a thick wall finite element model of the human carotid bifurcation. J. Biomech..

[bib13] Eliasziw M., Streifler J.Y., Fox A.J., Hachinski V.C., Ferguson G.G., Barnett H.J. (1994). Significance of plaque ulceration in symptomatic patients with high-grade carotid stenosis. North American symptomatic carotid endarterectomy trial. Stroke.

[bib14] Gao H., Long Q. (2008). Effects of varied lipid core volume and fibrous cap thickness on stress distribution in carotid arterial plaques. J. Biomech..

[bib15] Gao H., Long Q., Das S.K., Sadat U., Graves M., Gillard J.H., Li Z.Y. (2011). Stress analysis of carotid atheroma in transient ischemic attack patients: evidence for extreme stress-induced plaque rupture. Ann. Biomed. Eng..

[bib16] Holzapfel G.A., Mulvihill J.J., Cunnane E.M., Walsh M.T. (2014). Computational approaches for analyzing the mechanics of atherosclerotic plaques: a review. J. Biomech..

[bib17] Huang X., Yang C., Zheng J., Bach R., Muccigrosso D., Woodard P.K., Tang D. (2014). Higher critical plaque wall stress in patients who died of coronary artery disease compared with those who died of other causes: a 3D fsi study based on ex vivo mri of coronary plaques. J. Biomech..

[bib18] Huang Y., Teng Z., Sadat U., Graves M.J., Bennett M.R., Gillard J.H. (2014). The influence of computational strategy on prediction of mechanical stress in carotid atherosclerotic plaques: comparison of 2D structure-only, 3D structure-only, one-way and fully coupled fluid-structure interaction analyses. J. Biomech..

[bib19] Kaazempur-Mofrad M., Younis H., Patel S., Isasi A., Chung C., Chan R., Hinton D., Lee R., Kamm R. (2003). Cyclic strain in human carotid bifurcation and its potential correlation to atherogenesis: idealized and anatomically-realistic models. J. Eng. Math..

[bib20] Kaazempur-Mofrad M.R., Younis H.F., Patel S., Isasi A., Chung C., Chan R.C., Hinton D.P., Lee R.T., Kamm R.D. (2003). Cyclic strain in human carotid bifurcation and its potential correlation to atherogenesis: idealized and anatomically-realistic models. J. Eng. Math..

[bib21] Khosla S., Morris D.R., Moxon J.V., Walker P.J., Gasser T.C., Golledge J. (2014). Meta-analysis of peak wall stress in ruptured, symptomatic and intact abdominal aortic aneurysms. Br. J. Surg..

[bib22] Lawlor M.G., O׳Donnell M.R., O׳Connell B.M., Walsh M.T. (2011). Experimental determination of circumferential properties of fresh carotid artery plaques. J. Biomech..

[bib23] Leach J.R., Rayz V.L., Soares B., Wintermark M., Mofrad M.R., Saloner D. (2010). Carotid atheroma rupture observed in vivo and fsi-predicted stress distribution based on pre-rupture imaging. Ann. Biomed. Eng..

[bib24] Lee R.T., Schoen F.J., Loree H.M., Lark M.W., Libby P. (1996). Circumferential stress and matrix metalloproteinase 1 in human coronary atherosclerosis implications for plaque rupture. Arterioscler. Thromb. Vasc. Biol..

[bib25] Li Z.Y., Howarth S., Trivedi R.A. (2006). Stress analysis of carotid plaque rupture based on in vivo high resolution mri. J. Biomech..

[bib26] Li Z.Y., Howarth S.P., Tang T., Graves M.J. (2007). Structural analysis and magnetic resonance imaging predict plaque vulnerability: a study comparing symptomatic and asymptomatic individuals. J. Vasc. Surg..

[bib27] Maher E., Creane A., Sultan S., Hynes N., Lally C., Kelly D.J. (2009). Tensile and compressive properties of fresh human carotid atherosclerotic plaques. J. Biomech..

[bib28] Mulvihill J.J., Cunnane E.M., McHugh S.M., Kavanagh E.G., Walsh S.R., Walsh M.T. (2013). Mechanical, biological and structural characterization of in vitro ruptured human carotid plaque tissue. Acta Biomater..

[bib29] Ogden R.W., Holzapfel G.A., Ogden R.W. (2003). Nonlinear elasticity, anisotropy, material stability and residual stresses in soft tissue. Biomechanics of Soft Tissue in Cardiovascular Systems..

[bib30] Ohayon J., Tracqui P. (2005). Computation of adherent cell elasticity for critical cell-bead geometry in magnetic twisting experiments. Ann. Biomed. Eng..

[bib31] Richardson P.D., Davies M.J., Born G.V. (1989). Influence of plaque configuration and stress distribution on fissuring of coronary atherosclerotic plaques. Lancet.

[bib32] Sadat U., Teng Z., Young V.E., Graves M.J., Gaunt M.E., Gillard J.H. (2011). High-resolution magnetic resonance imaging-based biomechanical stress analysis of carotid atheroma: a comparison of single transient ischaemic attack, recurrent transient ischaemic attacks, non-disabling stroke and asymptomatic patient groups. Eur. J. Vasc. Endovasc. Surg..

[bib33] Sadat U., Teng Z., Young V.E., Walsh S.R., Li Z.Y., Graves M.J., Varty K., Gillard J.H. (2010). Association between biomechanical structural stresses of atherosclerotic carotid plaques and subsequent ischaemic cerebrovascular events—a longitudinal in vivo magnetic resonance imaging-based finite element study. Eur. J. Vasc. Endovasc. Surg..

[bib34] Sadat U., Weerakkody R.A., Bowden D.J., Young V.E., Graves M.J., Li Z.Y., Tang T.Y., Gaunt M.E., Hayes P.D., Gillard J.H. (2009). Utility of high resolution mr imaging to assess carotid plaque morphology: a comparison of acute symptomatic, recently symptomatic and asymptomatic patients with carotid artery disease. Atherosclerosis.

[bib35] Singh N., Moody A.R., Gladstone D.J., Leung G., Ravikumar R., Zhan J., Maggisano R. (2009). Moderate carotid artery stenosis: MR imaging-depicted intraplaque hemorrhage predicts risk of cerebrovascular ischemic events in asymptomatic men. Radiology.

[bib36] Takaya N., Yuan C., Chu B., Saam T., Underhill H., Cai J., Tran N., Polissar N.L., Isaac C., Ferguson M.S., Garden G.A., Cramer S.C., Maravilla K.R., Hashimoto B., Hatsukami T.S. (2006). Association between carotid plaque characteristics and subsequent ischemic cerebrovascular events: aprospective assessment with mri—initial results. Stroke.

[bib37] Tang D., Teng Z., Canton G., Yang C., Ferguson M., Huang X., Zheng J., Woodard P.K., Yuan C. (2009). Sites of rupture in human atherosclerotic carotid plaques are associated with high structural stresses: an in vivo mri-based 3D fluid-structure interaction study. Stroke.

[bib38] Tang D., Yang C., Canton G., Wu Z., Hatsukami T., Yuan C. (2013). Correlations between carotid plaque progression and mechanical stresses change sign over time: a patient follow up study using mri and 3D fsi models. Biomed. Eng. Online.

[bib39] Tang D., Yang C., Kobayashi S., Zheng J., Woodard P.K., Teng Z., Billiar K., Bach R., Ku D.N. (2009). 3D mri-based anisotropic fsi models with cyclic bending for human coronary atherosclerotic plaque mechanical analysis. J Biomech Eng.

[bib40] Tang T., Howarth S., Li Z., Miller S., Graves M. (2008). Correlation of carotid atheromatous plaque inflammation with biomechanical stress: utility of uspio enhanced mr imaging and finite element analysis. Atherosclerosis..

[bib41] Teng Z., Brown A.J., Calvert P.A., Parker R.A., Obaid D.R., Huang Y., Hoole S.P., West N.E., Gillard J.H., Bennett M.R. (2014). Coronary plaque structural stress is associated with plaque composition and subtype and higher in acute coronary syndrome: the beacon I (biomechanical evaluation of atheromatous coronary arteries) study. Circ. Cardiovasc. Imaging.

[bib42] Teng Z., Feng J., Zhang Y., Huang Y., Sutcliffe M.P.F., Brown A.J., Jing Z., Gillard J.H., Lu Q. (2015). Layer- and direction-specific material properties, extreme extensibility and ultimate material strength of human abdominal aorta and aneurysm: a uniaxial extension study. Ann. Biomed. Eng..

[bib43] Teng Z., He J., Sadat U., Mercer J.R., Xiaoyan W., Bahaei N.S., Thomas O.M., Gillard J.H. (2014). How does juxtaluminal calcium affect critical mechanical conditions in carotid atherosclerotic plaque? An exploratory study. IEEE Trans. Biomed. Eng..

[bib44] Teng Z., Sadat U., Huang Y., Young V.E., Graves M.J., Lu J., Gillard J.H. (2011). In vivo mri-based 3D mechanical stress–strain profiles of carotid plaques with juxtaluminal plaque haemorrhage: an exploratory study for the mechanism of subsequent cerebrovascular events. Eur. J. Vasc. Endovasc. Surg..

[bib45] Teng Z., Sadat U., Wang W., Bahaei N.S., Chen S., Young V.E., Graves M.J., Gillard J.H. (2013). Intraplaque stretch in carotid atherosclerotic plaque—an effective biomechanical predictor for subsequent cerebrovascular ischemic events. PLoS One.

[bib46] Teng Z., Zhang Y., Huang Y., Feng J., Yuan J., Lu Q., Sutcliffe M.P.F., Brown A.J., Jing Z., Gillard J.H. (2014). Material properties of components in human carotid atherosclerotic plaques: a uni-axial extension study. Acta Biomater..

[bib47] Versluis A., Bank A.J., Douglas W.H. (2006). Fatigue and plaque rupture in myocardial infarction. J. Biomech..

[bib48] Zheng, H., 2008. On the predictive capability and stability of rubber material models. (Master thesis).

[bib49] Zhu C., Teng Z., Sadat U., Young V.E., Graves M.J., Li Z.Y., Gillard J.H. (2010). Normalized wall index specific and mri-based stress analysis of atherosclerotic carotid plaques: a study comparing acutely symptomatic and asymptomatic patients. Circ. J..

