# Supplementary material for: The influence of constitutive law choice used to characterise atherosclerotic tissue material properties on computing stress values in human carotid plaques
Source: J Biomech. 2015 Nov 5;48(14):3912–21. doi: 10.1016/j.jbiomech.2015.09.023 (PMC4655867; doi:10.1016/j.jbiomech.2015.09.023)
Supplement: Supplementary file 1 — Supplementary material [file mmc1.doc]

**Supplementary Material**

Supplement to: Teng Z, et al. **The influence of constitutive law choice used to characterise atherosclerotic tissue material properties on computing stress values in human carotid plaques**

For each tissue strip, a series of data points, ($\lambda_{j}, \sigma_{j}$), was obtained. The elastic energy at a certain stretch level, $\lambda_{J}$, was defined as,

$$W\left( \lambda_{J} \right)=\sum_{j=1}^{j=J} \sigma_{j}\Delta\lambda_{j}, \Delta\lambda_{j}=\frac{\lambda_{j+1}-\lambda_{j-1}}{2}$$

For tissue strip *k*, 100 equal distance intervals were placed between maximum [max($W_{k}(\lambda_{i})$] and minimum [min($W_{k}(\lambda_{i})$] energy levels. Stretch and stress within each energy interval from the same tissue type were averaged as shown in Figure S1. To avoid bias, intervals with at least 5 data points from different tissue strips were used for further analysis.

**
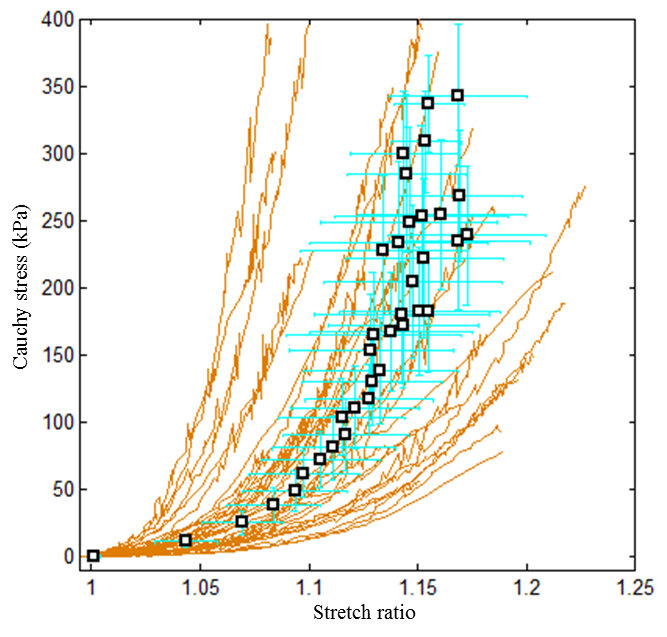
**

Figure S1. The stretch-stress curve of each media strip and the corresponding

averaged data points (black points) with error bars

**
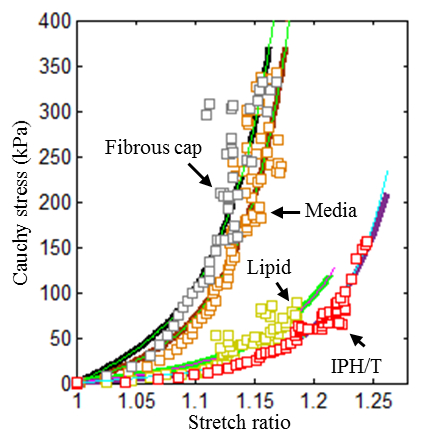
**

Figure S2. Two different sets of material constants of modified Mooney-Rivlin model could both characterize the stress-stretch data points of each atherosclerotic tissue (Thickness lines were produced using the constants listed in Table 1 in the manuscript and thin lines were from the other sets listed in Table 2 in the manuscript)

**
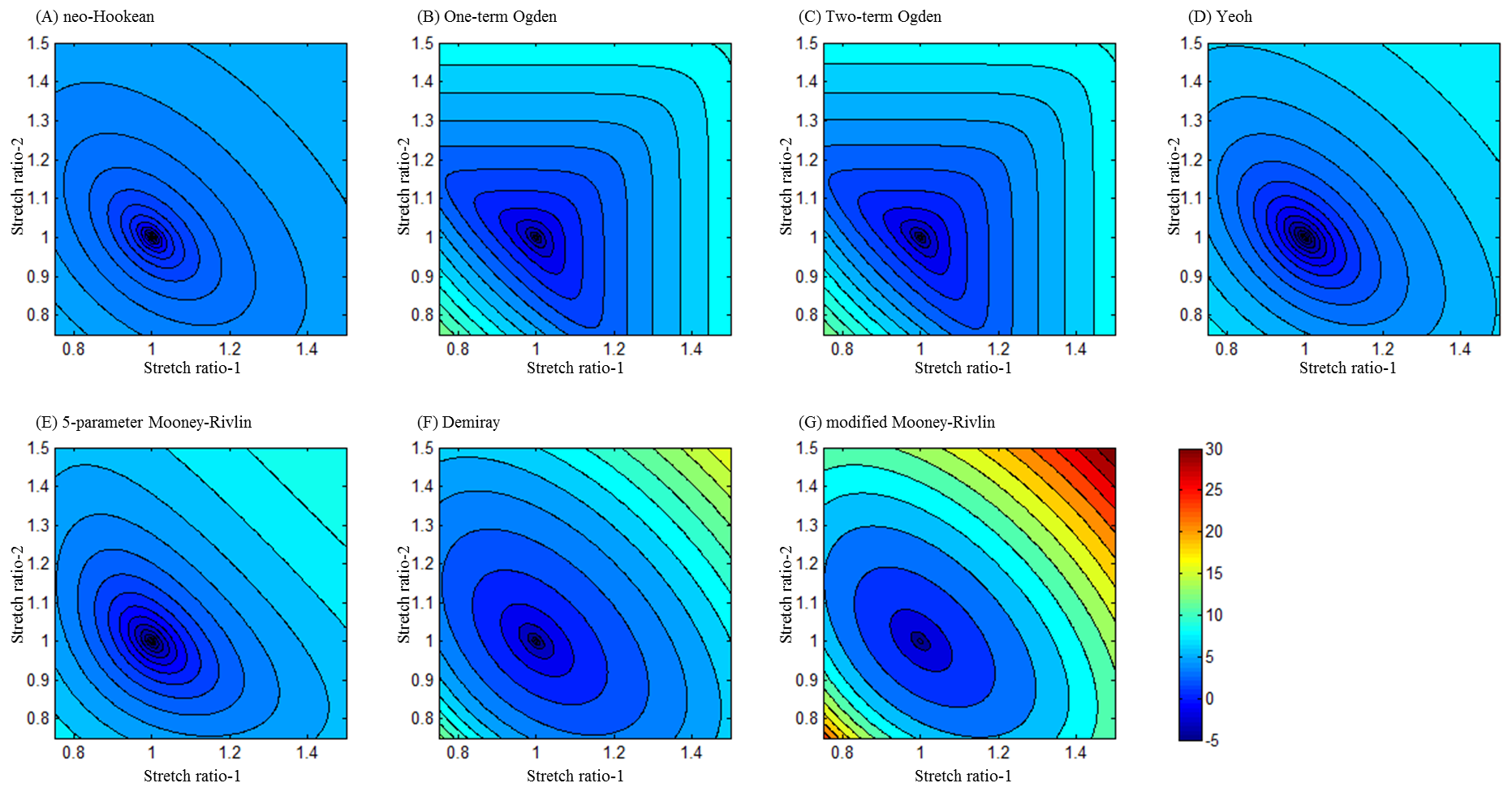
**

Figure S3. The logarithmized energy contours of media with different strain energy density functions

**
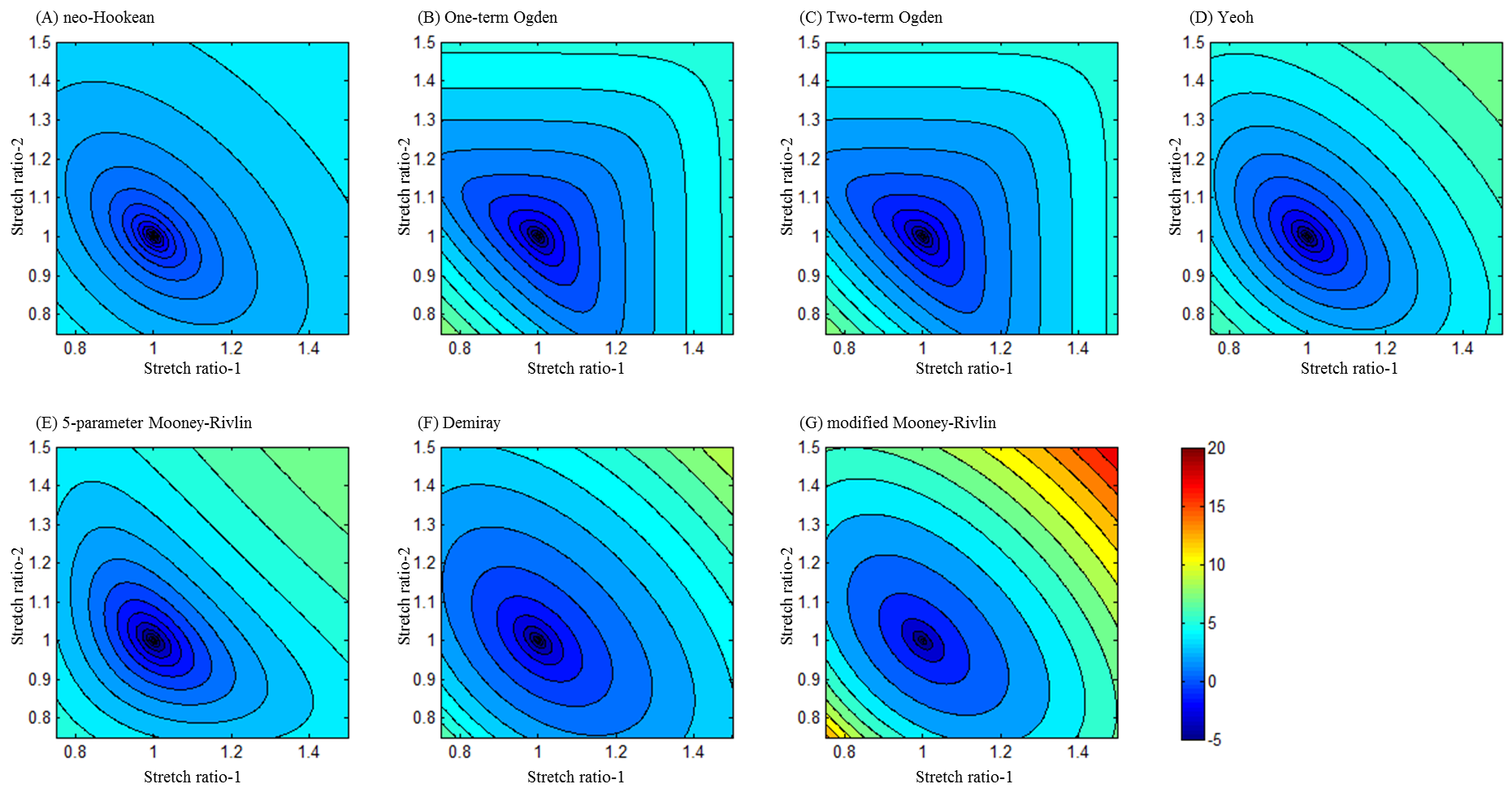
**

Figure S4. The logarithmized energy contours of lipid with different strain energy density functions


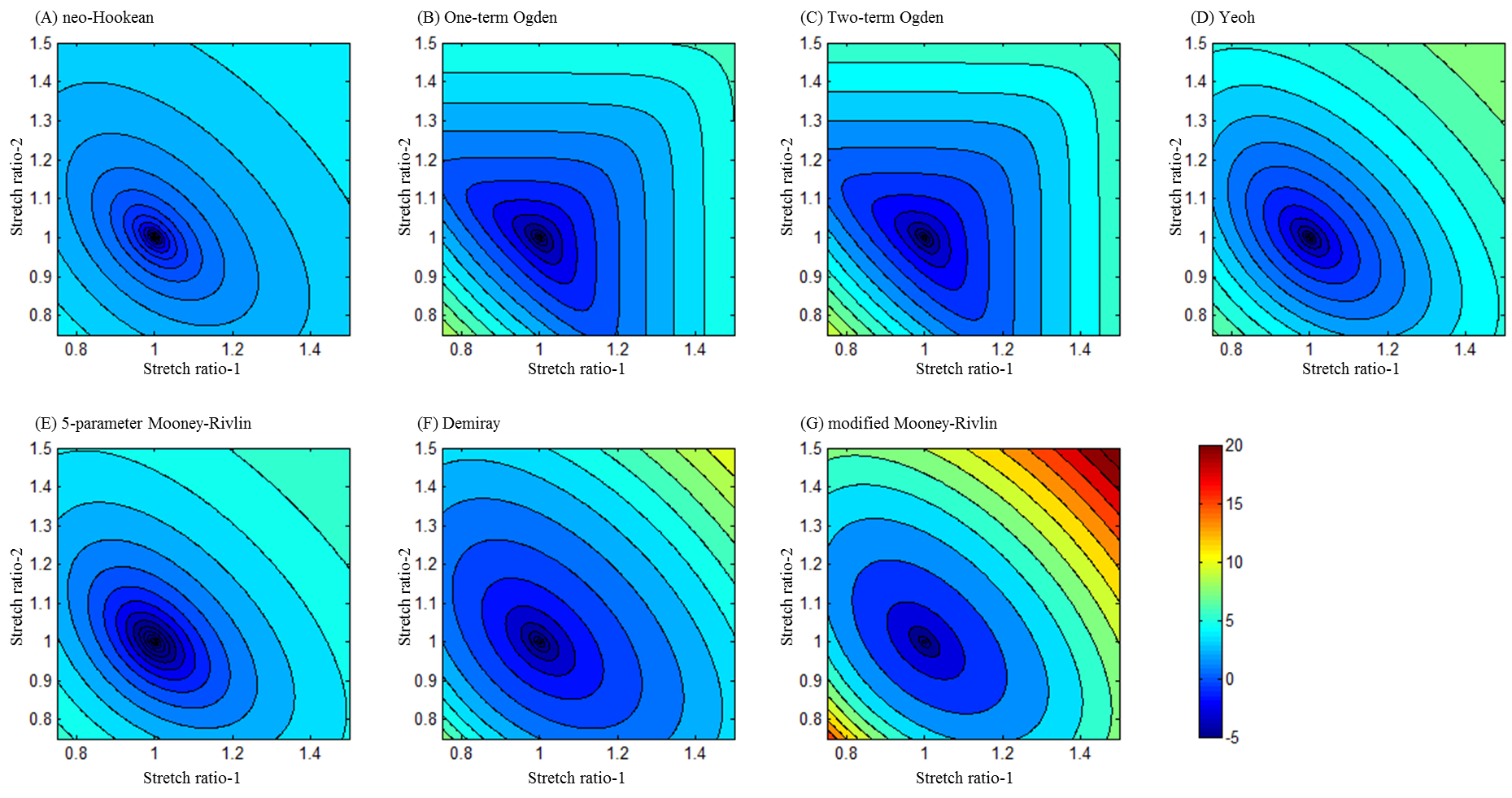


Figure S5. The logarithmized energy contours of intraplaque hemorrhage/thrombus with different strain energy density functions


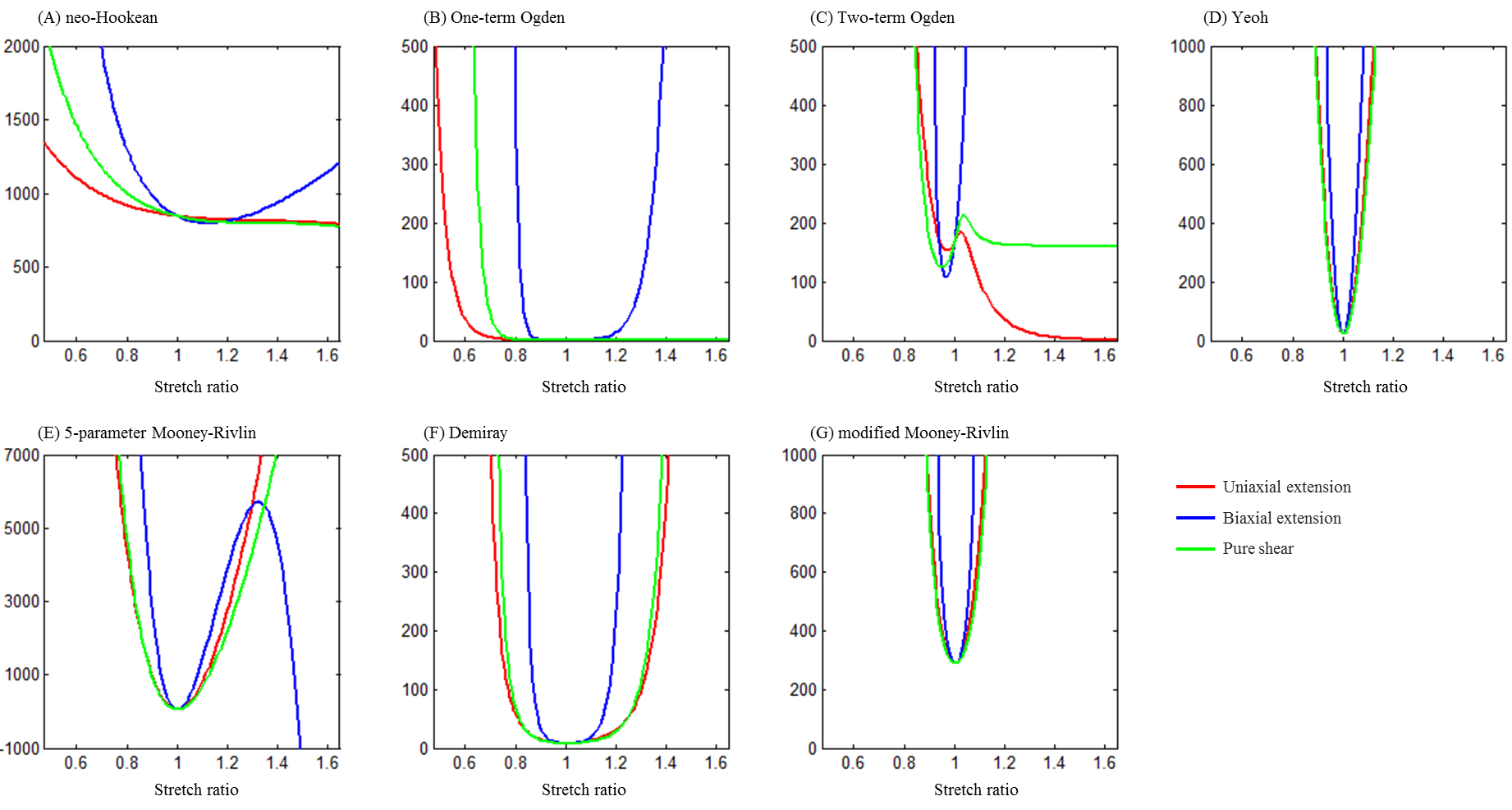


Figure S6. The stability curves of uniaxial extension, biaxial extension and pure shear of media with different strain energy density functions


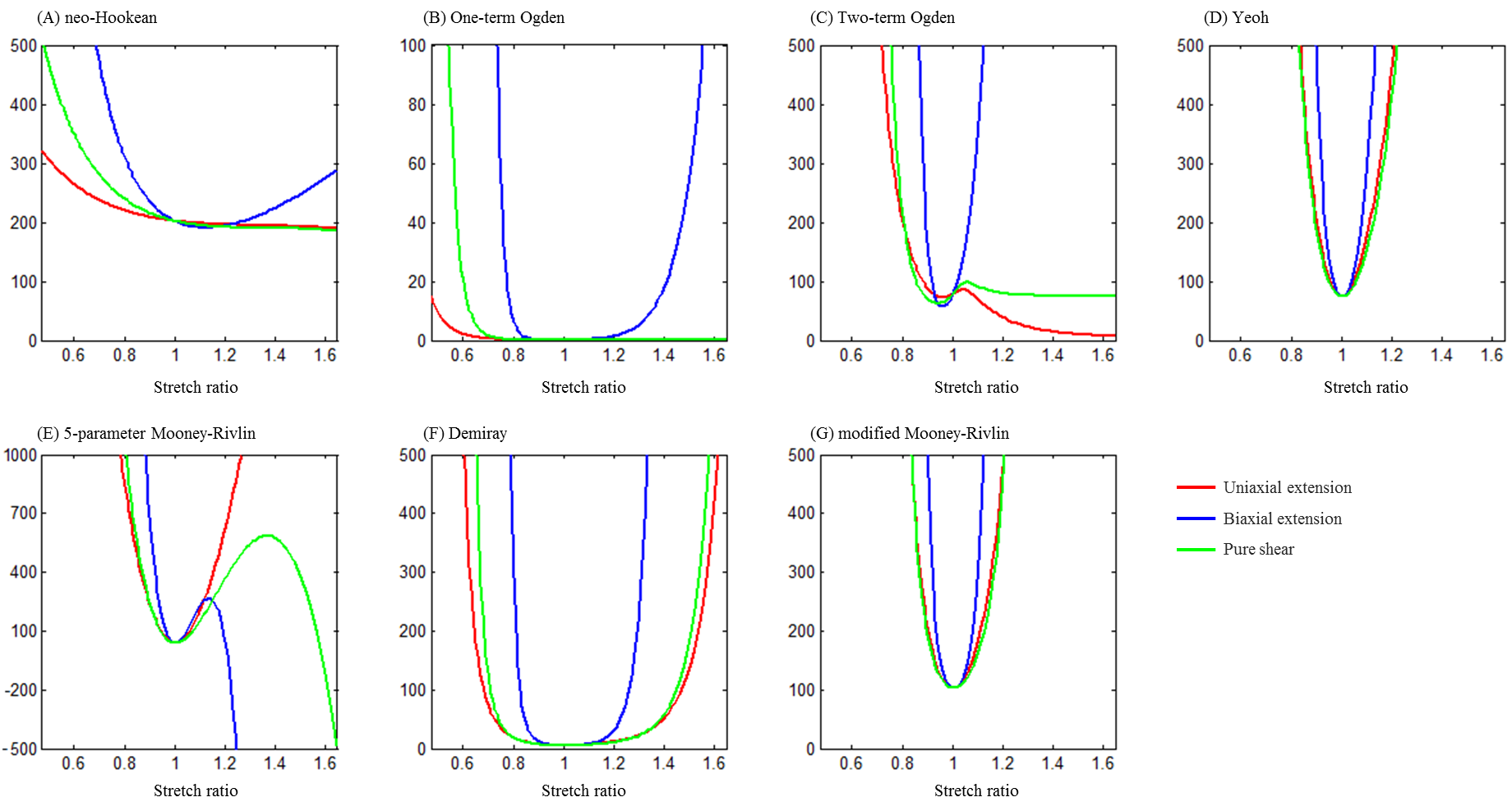


Figure S7. The stability curves of uniaxial extension, biaxial extension and pure shear of lipid with different strain energy density functions


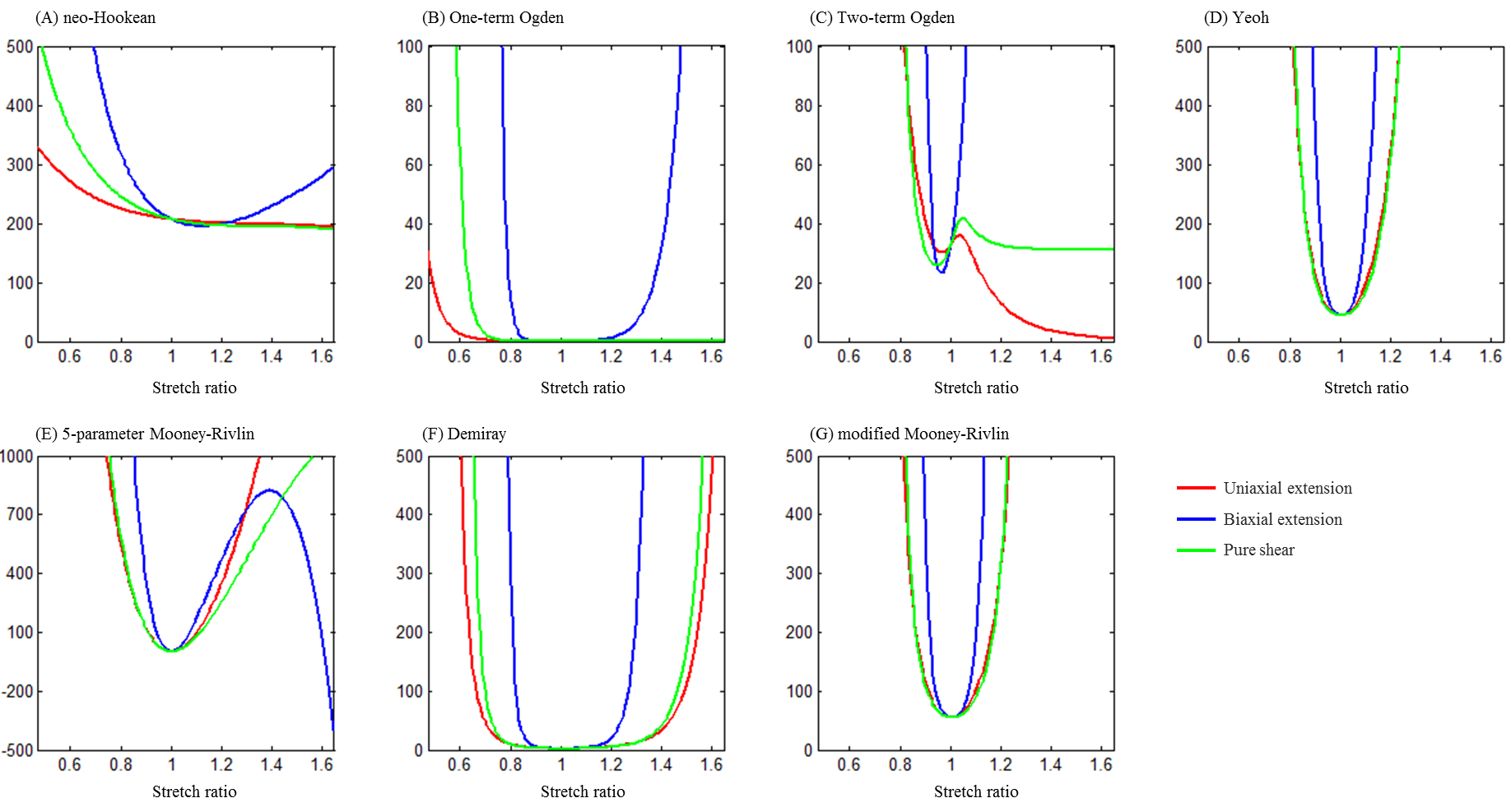


Figure S8. The stability curves of uniaxial extension, biaxial extension and pure shear of intraplaque hemorrhage/thrombus with different strain energy density functions
